# Supplementary material for: Reconstruction of the ancient cyanobacterial proto-circadian clock system KaiABC
Source: EMBO J. 2025 Apr 10;44(10):3025–46. doi: 10.1038/s44318-025-00425-0 (PMC12084410; doi:10.1038/s44318-025-00425-0)
Supplement: Supplementary file 1 — Table EV1 [file 44318_2025_425_MOESM1_ESM.docx]

**Table EV1. Summary of model refinement and validation statistics of anKaiC.**

| **Composition** | | **Parameters** |
| --- | --- | --- |
| **Chains** | 6 | |
| **Non-hydrogen atoms** | 45,942 | |
| **Residues** | 2,910 | |
| **Bonds (RMSD)** | | |
| **Length (Å)** | 0.003 | |
| **Angles (°)** | 0.577 | |
| **MolProbity score** | 1.36 | |
| **Clash score** | 6.60 | |
| **Ramachandran plot** | | |
| **Outliers (%)** | 0.00 | |
| **Allowed (%)** | 1.86 | |
| **Favored (%)** | 98.14 | |
| **Rotamer outliers (%)** | 0.96 | |
| **Cβ outliers (%)** | 0.00 | |
| **Model vs. Data** | | |
| **CC (mask)** | 0.77 | |
| **CC (volume)** | 0.70 | |
