# Supplementary material for: Reconstruction of the ancient cyanobacterial proto-circadian clock system KaiABC
Source: EMBO J. 2025 Apr 10;44(10):3025–46. doi: 10.1038/s44318-025-00425-0 (PMC12084410; doi:10.1038/s44318-025-00425-0)
Supplement: Supplementary file 2 — Appendix [file 44318_2025_425_MOESM2_ESM.docx]

**APPENDIX**

**Reconstruction of the ancient cyanobacterial proto-circadian clock system KaiABC**

Silin Li ^1,10^, Zengxuan Zhou ^1,10^, Yufeng Wan ^1,9,10^, Xudong Jia ^1,10^, Peiliang Wang ^1^, Yu Wang^2^, Taisen Zuo ^3,4^, He Cheng ^3,4^, Xiaoting Fang ^1^, Shuqi Dong ^5^, Jun He ^5^, Yilin Yang ^1^, Yichen Xu ^1^, Shaoxuan Fu ^1^, Xujing Wang ^1^, Ximing Qin ^6^, Qiguang Xie ^2^, Xiaodong Xu ^2^, Yuwei Zhao^7^, Dan Liang ^8^, Peng Zhang ^8^, Qinfen Zhang ^1^ & Jinhu Guo ^1✉^

^1^School of Life Sciences, Key Laboratory of Gene Engineering of the Ministry of Education, Sun Yat-sen University, Guangzhou, China. ^2^State Key Laboratory of Crop Stress Adaptation and Improvement, School of Life Sciences, Henan University, Kaifeng, China. ^3^Institute of High Energy Physics, Chinese Academy of Sciences, Beijing, China. ^4^Spallation Neutron Source Science Center, Dongguan, China. ^5^Center for Biomedical Digital Science, State Key Laboratory of Respiratory Disease, Guangdong Provincial Key Laboratory of Stem Cell and Regenerative Medicine, GIBH-CUHK Joint Research Laboratory on Stem Cell and Regenerative Medicine, Guangzhou Institutes of Biomedicine and Health, Chinese Academy of Sciences, Guangzhou, China. ^6^Department of Health Sciences, Institutes of Physical Science and Information Technology, Anhui University, Hefei, China. ^7^School of Life Sciences, Northwest University, Xi’an, China. ^8^State Key Laboratory of Biocontrol, Guangdong Provincial Key Laboratory for Aquatic Economic Animals, School of Life Sciences, Sun Yat-Sen University, Guangzhou, China. ^9^Present address: Department of Biology, Texas A&M University, College Station, TX, USA. ^10^These authors contributed equally: Silin Li, Zengxuan Zhou, Yufeng Wan, Xudong Jia.

^🖂^Co‐correspondence: [guojihhu@mail.sysu.edu.cn](mailto:guojihhu@mail.sysu.edu.cn) (J.G.)

**TABLE OF CONTENTS**

**Appendix Figure S1:**SDS-polyacrylamide gel electrophoresis of purified prokaryotically expressed KaiABC and anKaiABC proteins page ...............................2

**Appendix Figure S2:**SDS-polyacrylamide gel electrophoresis of purified prokaryotically expressed KaiC mutant proteins ...................................................page 3

**

**

**Appendix Figure S1. SDS-polyacrylamide gel electrophoresis of purified prokaryotically expressed KaiABC and anKaiABC proteins.** (**A-C**) SDS-polyacrylamide gel electrophoresis of purified KaiABC proteins. (**D-F**) SDS-polyacrylamide gel electrophoresis of purified KaiABC proteins. (**E**) SDS-polyacrylamide gel electrophoresis of anKaiB proteins purified with GSTrap columns once more after purification with GSTrap columns. (**G,H)** SDS-polyacrylamide gel electrophoresis of purified Tobacco Etch Virus protease (TEVp) (**G**), and PreScission Protease (PSP) (**H**) proteins. PSP was used to eliminate the MBP fusion tag and TEVp was used to remove the GST fusion tag. In (**A**,**C**,**D** and **F**), Q columns were used for purification; in (**B**,**E** and **H**), GSTrap columns were used for purification; in (**G**), Ni-NTA columns were used for protein purification. T: total cell lysates; S: supernatant; F: flow-through fractions; W1/2, fractions of wash 1/2; M, protein marker; E1-E9, elution fractions; Q1/2, proteins obtained by purification and following hyperfiltration concentration. All the purity values of these purified proteins were > 90%, which were measured and assessed with NIH ImageJ analysis software (version 1.51).

**

**

**Appendix Figure S2. SDS-polyacrylamide gel electrophoresis of purified prokaryotically expressed KaiC mutant proteins.** (**A-G**) SDS-polyacrylamide gel electrophoresis of different purified proteins KaiC-V118A proteins (**A**,**B**), KaiC-S229T (**C**,**D**), KaiC-T240N (**E**,**F**), and KaiC-S229T/T240N (**G**). In **A**-**G**: Q columns and GSTrap columns were used for purification. T: total cell lysates; Sup: supernatant; Sed: Sediment; flow: flow-through fractions; W1/2, fractions of wash 1/2; E: eluted proteins; M: protein marker; Qin, Q-column inflow liquid; Qout, Q-column effluent. As shown in panel **A**, the purified products contained unspecific proteins after passing through GST-column. The purified products were subsequently passed through Q-column for further purification and enrichment (**B**). All the purity values of these purified proteins were > 90%, which were measured and assessed with NIH ImageJ analysis software (version 1.51).
