# Supplementary material for: Reconstruction of the ancient cyanobacterial proto-circadian clock system KaiABC
Source: EMBO J. 2025 Apr 10;44(10):3025–46. doi: 10.1038/s44318-025-00425-0 (PMC12084410; doi:10.1038/s44318-025-00425-0)
Supplement: Supplementary file 4 — Expanded View Figures [file 44318_2025_425_MOESM4_ESM.pdf]

Expanded View Figures

Figure EV1. Cryo-EM analysis of the anKaiC.

(**A**) Representative cryo-EM micrograph of anKaiC. (**B**) Fourier shell correlation curve of the anKaiC reconstruction. (**C**) Local resolution map of anKaiC reconstruction. (**D**) Image processing pipeline for anKaiC. Selected classes in each 3D classification are labelled by dotted line boxes. (**E**) Angular distribution of particle orientations used in the final reconstruction. (**F**) Conserved structure of partial A-loop between anKaiC (coloured in blue) and KaiC (PDB: 7S67, coloured in pink green). (**G**) The inside surface of anKaiC showing similarity to that of KaiC WT (PDB: 7S67). The surface (inside view) with identical amino acid residues in both anKaiC and KaiC are coloured in grey, the different amino acids between anKaiC and KaiC with hydrophilic residues are shown in cyan, and hydrophobic residues are shown in golden rod.

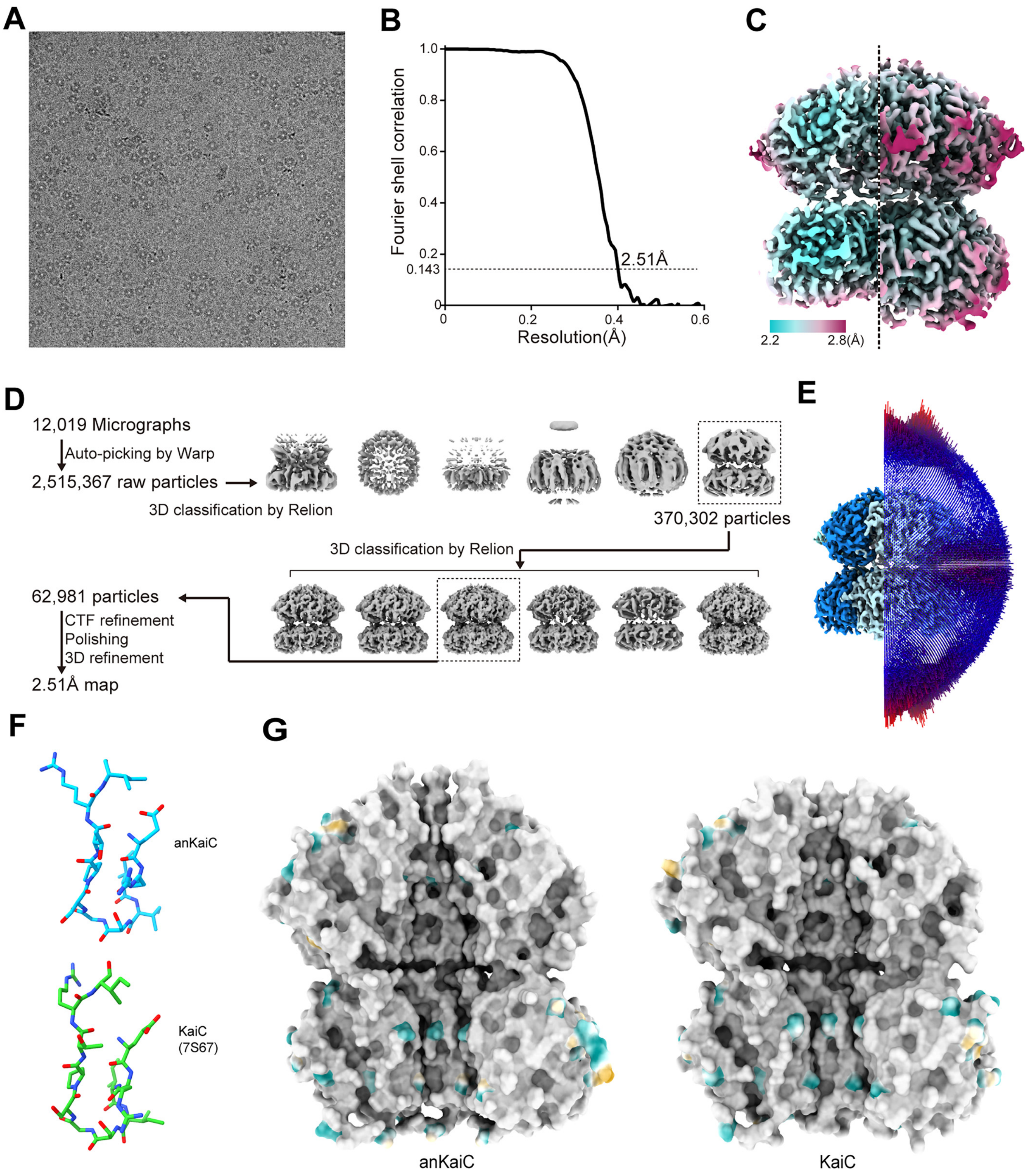
A
B
1.0
0.8
0.6
0.4
0.2
0.143
0
Fourier shell correlation
2.51Å
0 0.2 0.4 0.6
Resolution(Å)
C
2.2 2.8(Å)
D
12,019 Micrographs
Auto-picking by Warp
2,515,367 raw particles
3D classification by Relion
370,302 particles
3D classification by Relion
62,981 particles
CTF refinement
Polishing
3D refinement
2.51Å map
E
F
anKaiC
KaiC
(7S67)
G
anKaiC
KaiC

 © The Author(s)

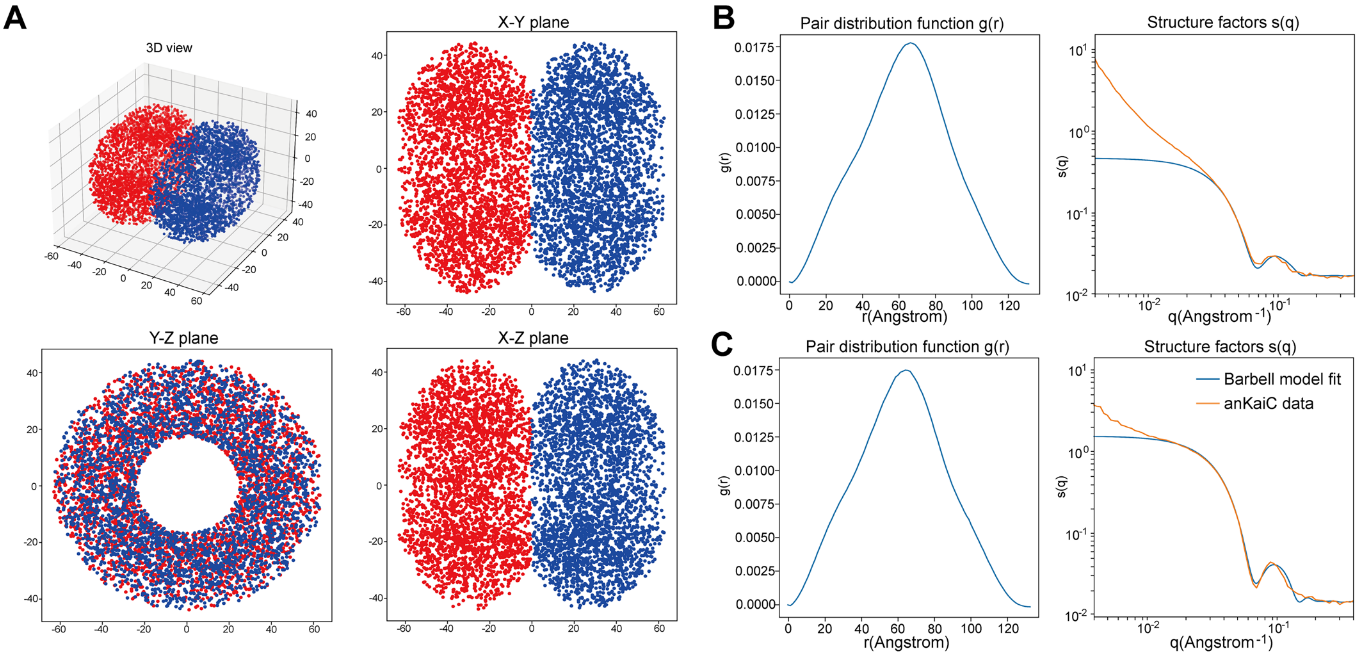

Figure EV2. Modelling of anKaiC and KaiC SANS data.

(**A**) The 3D and X, Y, Z projection of the dumbbell model of KaiC or anKaiC. (**B**) The pair distribution function (PDF) of the dumbbell model of KaiC and the best fit to the data with the dumbbell model. (**C**) The PDF of the dumbbell model of anKaiC and the best fit to the data.

© The Author(s)

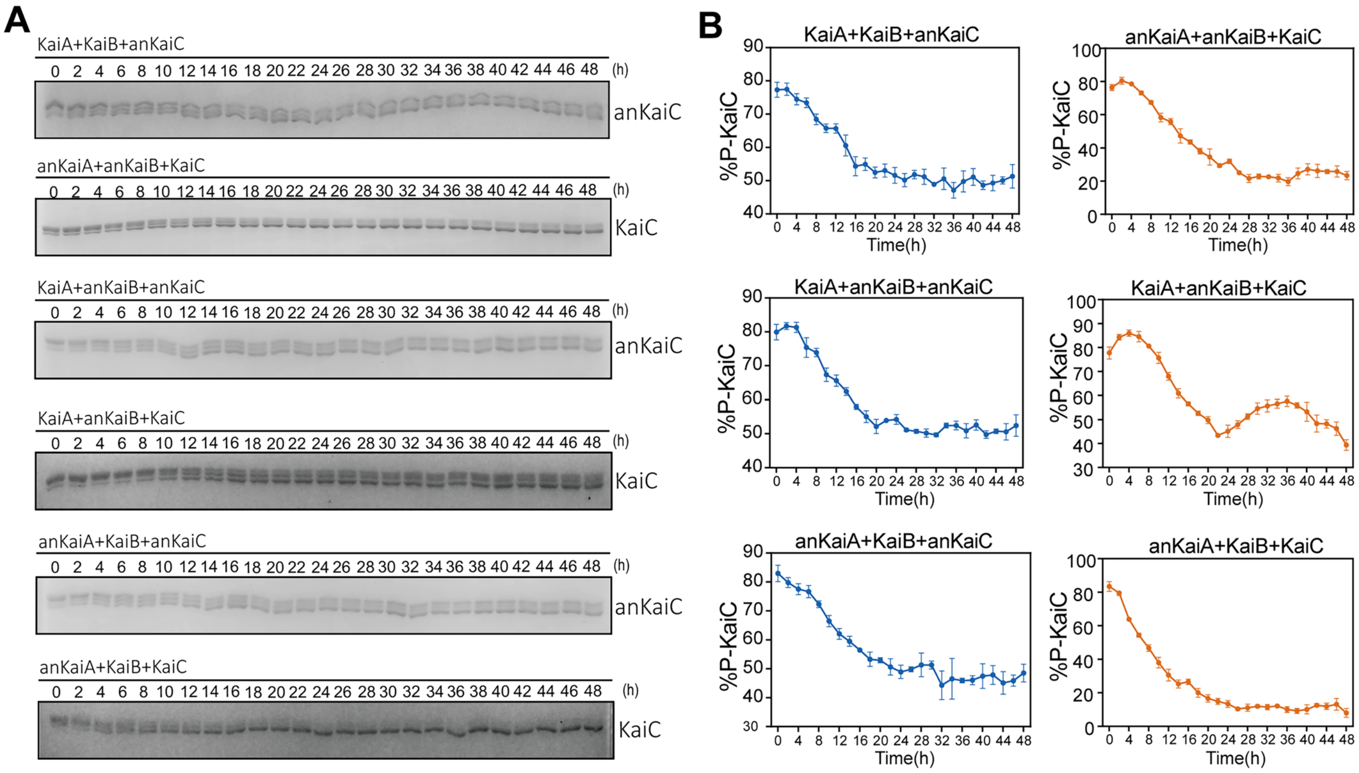

Figure EV3. Analysis of in vitro and in vivo rhythmicities in strains expressing different combinations of KaiABC proteins.

(**A**) In vitro phosphorylation of anKaiC/KaiC proteins in different combinations. Representative results of Coomassie brilliant blue staining are shown. (**B**) Statistical results of bioluminescence rhythms of the indicated combinations. The percentage of hyperphosphorylated KaiC was calculated. Data are means ± SD. *n* = 3. Source data are available online for this figure.

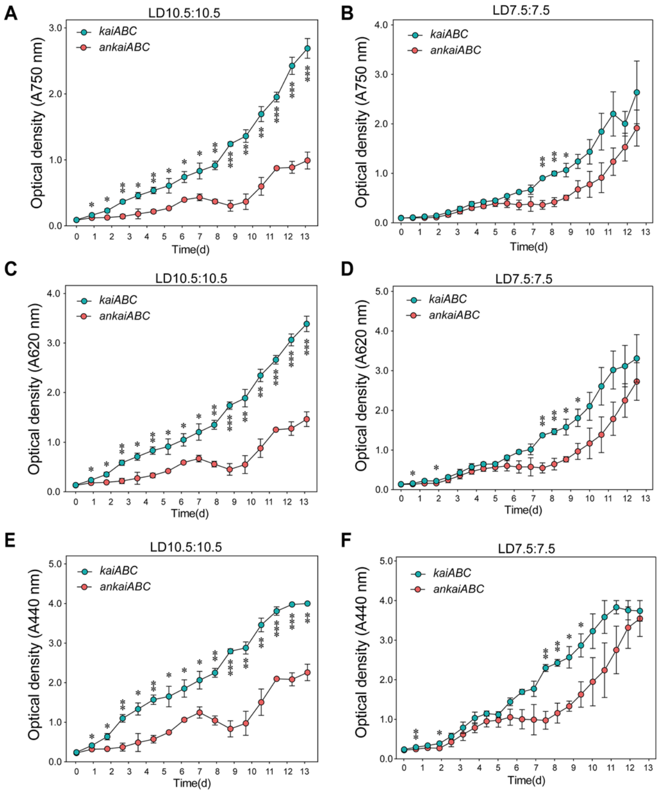

Figure EV4. Curves of growth, phycobilin and chlorophyll a content under LD10.5:10.5 and LD7.5:7.5 conditions.

(**A**, **B**) Growth curves of *ankaiABC* and *kaiABC* strains measured by light absorption at 750 nm under LD10.5:10.5 and LD7.5:7.5 conditions. In (**A**): *P* values from left to right: **P* = 0.0140, **P* = 0.0229, ***P* = 0.0065, **P* = 0.0346, ***P* = 0.0047, **P* = 0.0374, **P* = 0.0147, **P* = 0.0377, ***P* = 0.0016, ****P* = 0.0004, ***P* = 0.0028, ***P* = 0.0034, ****P* = 0.0001, ****P* = 0.0006, ****P* = 0.0010. In (**B**): *P* values from left to right: ***P* = 0.0037, ***P* = 0.0049, **P* = 0.0187. (**C**, **D**) Phycobilin levels of *ankaiABC* and *kaiABC* strains measured by light absorption at 620 nm under LD10.5:10.5 and LD7.5:7.5 conditions. In (**C**): *P* values from left to right: **P* = 0.0193, **P* = 0.0245, ***P* = 0.0077, **P* = 0.0367, ***P* = 0.0045, **P* = 0.0356, **P* = 0.0215, **P* = 0.0391, ***P* = 0.0017, ****P* = 0.0008, ***P* = 0.0062, ***P* = 0.0029, ****P* < 0.0001, ****P* = 0.0006, ****P* = 0.0009. In (**D**): *P* values from left to right: **P* = 0.0420, **P* = 0.0465, ***P* = 0.0034, ***P* = 0.0074, **P* = 0.0185, **P* = 0.0466. (**E**, **F**) Chlorophyll a levels of *ankaiABC* and *kaiABC* strains measured by light absorption at 440 nm under LD10.5:10.5 and LD7.5:7.5 conditions. In (**E**): *P* values from left to right: **P* = 0.0283, **P* = 0.0200, ***P* = 0.0063, **P* = 0.0367, ***P* = 0.0029, **P* = 0.0251, **P* = 0.0241, **P* = 0.0361,***P* = 0.0017, ****P* = 0.0008, ***P* = 0.0050, ***P* = 0.0064, ****P* < 0.0001, ****P* = 0.0003, ***P* = 0.0011. In (**F**): *P* values from left to right: ***P* = 0.0056, **P* = 0.0402, ***P* = 0.0054, ***P* = 0.0087, **P* = 0.0146, **P* = 0.0464. Data are means ± SE. *n* = 3 (independent experiments). Two-tailed unpaired Student's *t* test. Source data are available online for this figure.

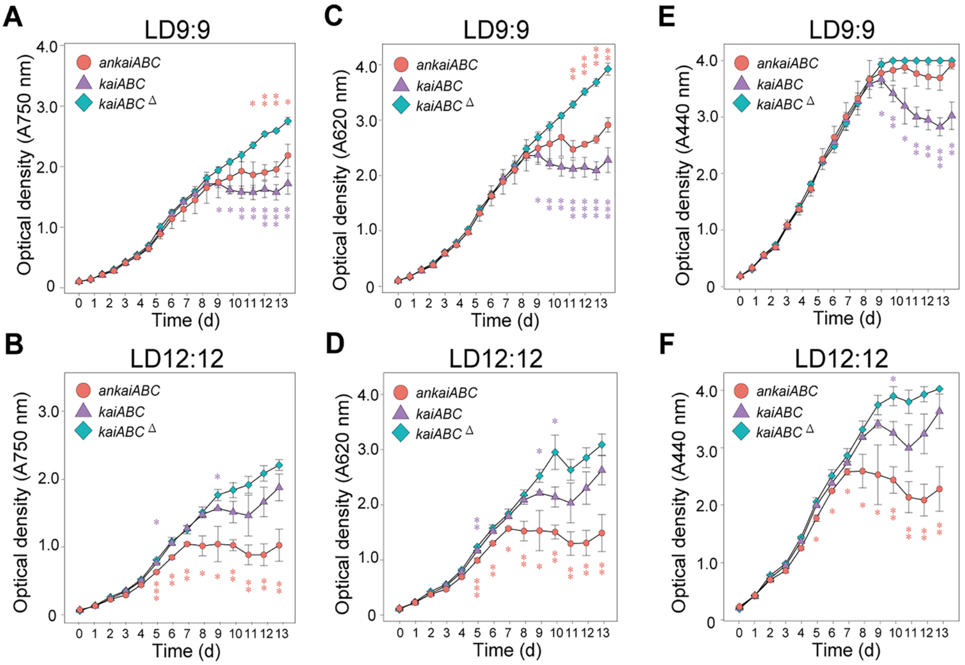

Figure EV5. Growth curves of cyanobacterial strains.

(**A**, **B**) Growth curves in LD9:9 and LD12:12 measured by light absorption at 750 nm. In (**A**): *P* values in purple from left to right: **P* = 0.0202, **P* = 0.0184, ***P* = 0.0015, ***P* = 0.0010, ****P* = 0.0008, ****P* = 0.0004, ***P* = 0.0014; *P* values in red from left to right: **P* = 0.0348, ***P* = 0.0024, ***P* = 0.0023, **P* = 0.0145. In (**B**): *P* values in purple from left to right: **P* = 0.0100, **P* = 0.0282; *P* values in red from left to right: ****P* = 0.0002, ***P* = 0.0060, ***P* = 0.0085, **P* = 0.0132, **P* = 0.0199, ***P* = 0.0012, ***P* = 0.0016, ***P* = 0.0010, ***P* = 0.0026. (**C**, **D**) Phycobilin content measured by light absorption at 620 nm. In (**C**): *P* values in purple from left to right: **P* = 0.0381, ***P* = 0.0013, ***P* = 0.0015, ****P* = 0.0007, ****P* = 0.0005, ****P* = 0.0002, ****P* = 0.0007; *P* values in red from left to right: ***P* = 0.0024, ****P* < 0.0001, ****P* = 0.0002, ***P* = 0.0010. In (**D**): *P* values in purple from left to right: ***P* = 0.0023, **P* = 0.0216, **P* = 0.0338; *P* values in red from left to right: ****P* = 0.0009, ***P* = 0.0034, **P* = 0.0111, ***P* = 0.0095, **P* = 0.0212, ***P* = 0.0037, ***P* = 0.0027, ***P* = 0.0016, ***P* = 0.0042. (**E**, **F**) Content of chlorophyll a measured by light absorption at 440 nm. (**A–F**). In (**E**): *P* values in purple from left to right: **P* = 0.0171, ***P* = 0.0048, **P* = 0.0227, ***P* = 0.0013, ***P* = 0.0010, ****P* = 0.0005, ***P* = 0.0047. In (**F**): *P* values in purple from left to right: **P* = 0.0255; *P* values in red from left to right: **P* = 0.0112, **P* = 0.0265, **P* = 0.0395, **P* = 0.0350, **P* = 0.0347, ***P* = 0.0019, ***P* = 0.0023, ***P* = 0.0010, ***P* = 0.0032. Data are means ± SD, *n* = 3 (independent experiments). Asterisks in purple denote significance between *kaiABC* and *kaiABC*$^{\Delta}$, asterisks in red denote significance between *ankaiABC* and *kaiABC*$^{\Delta}$. Two-tailed unpaired Student's *t* test. Source data are available online for this figure.

 © The Author(s)
